# Authenticity as a Mediator of the Relationship Between Power Contingent Self-Esteem and Subjective Well-Being

**DOI:** 10.3389/fpsyg.2018.01066

**Published:** 2018-06-27

**Authors:** Yi’nan Wang, Ziyi Li

**Affiliations:** Beijing Key Laboratory of Applied Experimental Psychology, National Demonstration Center for Experimental Psychology Education, Faculty of Psychology, Beijing Normal University, Beijing, China

**Keywords:** power, contingent self-esteem, true self, well-being, authenticity

## Abstract

Drawing on Eastern wisdom and Self-Determination Theory (Deci and Ryan, 1995), the current study conceptualized a new form of maladaptive self-esteem, the power contingent self-esteem, which is extremely contingent on one’s sense of power, and posits it is related to low subjective well-being by making people experience less authenticity. In Study 1, we found that general power contingent self-esteem was consistently linked to low subjective well-being. More importantly, the negative relationship between power contingent self-esteem and subjective well-being was mediated by authenticity. Study 2 further confirmed the mediation effect between power contingent self-esteem role and satisfaction through authenticity across four different roles (work, romance, friendship, and parent–child relationships). The finding of the negative relationship between power contingent self-esteem and subjective well-being via authenticity contributes to understanding the complicated association between power, self-esteem, and life satisfaction.

## Introduction

Power considerations are omnipresent in human being’s everyday life (Keltner et al., 2003; Anderson et al., 2012). Accumulating evidence shows that the possession of power can lead to both higher self-esteem and better well-being (Adler et al., 2000; Wojciszke and Struzynska-Kujalowicz, 2007; Duguid and Goncalo, 2012; Wang, 2015a). However, we are still far from understanding the complicated relationship between power, self-esteem, and well-being.

Although high power is linked to high self-esteem (Wang, 2015a), once the individual’s self-esteem is contingent on his/her sense of power, it will have negative effects on his/her well-being according to Self-Determination Theory (SDT, Deci and Ryan, 1995). Furthermore, drawing on the research linking contingent self-esteem to decreased connection between internal needs and external performance, the current study proposed the power contingent self-esteem is associated with lower subjective well-being (SWB), which is defined as a person’s affective and cognitive appraisal of one’s life (Diener et al., 1985), through decreased authenticity.

In short, the main purpose of current study is to investigate whether and how power contingent self-esteem exerts effects on people’s SWB. Understanding these relationships will contribute to enhancing people’s well-being through knowing how to navigate the relation between power and self-esteem.

### Power, Self-Esteem, and Well-Being

In definition, power is the potential to influence and control others (Fiske, 1993; Keltner et al., 2003). Because people who have power can effectively exert influence on other people, which can lead them to have faith in that they are superior to other individuals, they usually have high self-esteem (Kipnis, 1972). This is termed global judgments of self-worth (Rosenberg, 1965). Although both power and self-esteem are robust predictors of well-being (Diener and Diener, 2009; Kifer et al., 2013; Moksnes and Espnes, 2013; Wang, 2015a,b), we still know little about how the complicated association between power and self-esteem influences individuals’ well-being.

Theoretically, the failure to exert power over others might lead to individuals disregard the ineffectual incident as irrelevant to their self-evaluation, or as an enormous hit to their self-esteem. According to previous studies on contingent self-esteem, once the individual’s self-evaluation is contingent on the failure of controlling others, they will experience a decrease in well-being (Kernis and Goldman, 2006; Knee et al., 2008).

### Contingent Self-Esteem Damages Well-Being

In 1890, William James suggested that people’s self-esteem waves around its typical level in answer to accomplishments and defeats in the domains in which one’s self-worth is at stake. Building on James’ insights, Crocker and her colleagues develop the conception of ‘contingent self-esteem’ to determine how, when, and why life affairs affect the self (Crocker and Wolfe, 2001; Crocker et al., 2003; Crocker and Knight, 2005). Firstly, people have individual differences in the contingencies on which they build their self-esteem, and this can be divided into two categories: external contingencies (e.g., social approval or competencies) and internal contingencies (e.g., God’s love). Secondly, a range of evidence shows that external contingency of self-worth (such as performance) is related to lower self-esteem (Crocker et al., 2003), reduced psychological well-being, and higher levels of daily stress and depression (Sargent et al., 2006; Wouters et al., 2013; Schöne et al., 2015).

By the same token, East Asian Buddhism also speculates that the acquisition of material goods, reputation, or power may give rise to happiness, but is unsustainable. When those stimulating source cease, the pleasure linked with them will wanes (Ricard, 2008). Therefore, to realize a state of sustainable happiness, people must not be contingent on the existence of pleasurable stimuli (Wallace, 1999; Wallace and Shapiro, 2006). Similarly, the SDT-based framework for well-being (Ryan et al., 2008) considers that health and living well are not supported by extrinsic goals or values (such as wealth or power), but rather by intrinsic goals or values (such as personal growth). SDT began with the distinction between intrinsic motivation and extrinsic motivation. Intrinsic motivation refers to engaging an activity for the inherent need of the activity itself, which represents a highly autonomous form of regulation. By contrast, extrinsic motivation is defined as performing an activity with the aim of achieving some result that represents external regulation, such as acting to avoid punishments or obtain contingent rewards. Then, the contingency leaves one vulnerable to both external social pressures and the chase of unrealizable goals that are followed by inauthentic living. For this reason, the action driven by esteem-related contingency become detrimental to well-being (Ryan and Brown, 2003).

Previous studies have examined the damaging influence of self-esteem contingent on kinds of domains, such as social acceptance (Leary and Baumeister, 2000), relationships (Knee et al., 2008), and academic competency, family support or appearance (Crocker and Wolfe, 2001). Although power has been put forward as an important basis of self-esteem for human beings (Coopersmith, 1967), there are surprisingly few studies on the self-esteem relying on power. To our knowledge, there is only one previous study has researched power contingent self-worth (Crocker et al., 2002). In Crocker et al. (2002)’s study, they proposed a measure of power contingency as a four-item subscale of contingencies of self-worth (e.g., “Being in a very powerful position would enhance my regard.”). While, they found power contingency had no association with academic competence, nor moderated the effects of acceptance/rejection on daily global self-esteem.

Considering power is an important basis of self-esteem, it is a matter of course that possessing power will give rise to people’s self-esteem. Accordingly, power contingent self-esteem will not necessarily lead to negative effects. While according to Chinese wisdom on middle way (or moderation) which think highly of too much is as bad as too little (*e.g.*, *guo you bu ji* and *wu ji bi fan*) (Cheung et al., 2003), when individuals extremely depend their self-esteem on power, they will be sensitive to small success/failure at having/losing power, which reflects unstable or fragile self-esteem (Kernis, 2003) and then shows negative effects on their well-being. Therefore, we predicted that when individual’s self-esteem is extremely contingent on his/her power, it will lower their SWB.

The current study will extend Crocker et al. (2002)’s study on power contingency with proposing a new type of power contingent self-esteem which assesses extent to which people extremely depend their self-esteem on power, such as very little lose of power leads to decreased self-esteem. Furthermore, we will examine whether and how the power contingent self-esteem will influence people’s well-being.

### Authenticity, Power Contingent Self-Esteem, and Well-Being

Different from previous study on power contingent self-worth, we conceptualized a new form of maladaptive self-esteem that is extremely contingent on one’s sense of power, called as Power Contingent Self-Esteem (PCSE). This represents a way in which the self is overly dependent on one’s power or ability to control others (Wang, 2017, unpublished). PCSE reflects the extent to which people’s self-regard overwhelmingly relies on the process and outcome of their influence on others. To someone who have higher PCSE, even minor powerless events might be a considerable blow to him/her because of the implications for their self-regard.

According to SDT (Deci and Ryan, 1995), contingent self-esteem might differ from genuine and high self-esteem. SDT argues that motivation for the contingencies of self-esteem fall on a spectrum from extrinsically motivated behavior—performed in the cause of the external rewards or punishments—and fully intrinsically motivated behavior (Deci and Ryan, 1995). Specifically, contingent self-esteem is extrinsically motivated and prioritizes external approval over inner appraisal. By contrast, true self-esteem, which refers to a sense of genuine self-worth, self-respect, and self-acceptance, despite a realistic recognition of their weakness and mistakes (Deci and Ryan, 1995), is intrinsically motivated and prioritizes inner values over others’ approval. Considering PCSE reflects a lack of feeling genuine validation, it would be negatively associated with authenticity, which refers to the individual behaving with a full sense of choice and self-expression in different situations (Ryan and Deci, 2000; Kernis, 2003; Kifer et al., 2013; Wang, 2014). Furthermore, considerable research indicates that authenticity is vital for promoting well-being (Goldman and Kernis, 2002; Wood et al., 2008; Wang, 2014, 2015a). Therefore, we hypothesized that PCSE would be negatively correlated with well-being through decreased authenticity.

### Overview of the Present Study

The present research examined whether and how PCSE is linked to SWB. Integrating evidence that contingent self-esteem hinders the expression of the real self, which is important for SWB, we hypothesized that PCSE decreases SWB through a lowered experience of authenticity. We examined this hypothesis in two studies. In Study 1, we measured dispositional PCSE, general authenticity, and SWB to examine whether general PCSE is negatively related to SWB through authenticity. In Study 2, we measured specific PCSE, authenticity, and role satisfaction in romantic-relationships, friendships, work, and parent–child relationships, to confirm whether the mediation effect of authenticity in the relationship between PCSE and SWB could be narrowed down to specific roles.

## Study 1

In Study 1, we examined the assumption that general PCSE would be a negative predictive variable of life satisfaction and that general self-authenticity would mediate the relationship between PCSE and life satisfaction.

### Participants

Two hundred and ten Chinese adults (Males = 91; Mean age = 31.90, *SD* = 7.43) were recruited from a professional website^1^ in April 2016. Participants were paid 10 RMB (about $1.5 U.S.) upon completion of the online questionnaires. Participants varied considerably in profession (e.g., 7% college students, 12% technical personnel, and 22% managerial personnel, and so on), socioeconomic status (monthly income from 1000 to above 20000 RMB), and education (junior middle school graduates 1%, high school graduates 8%, undergraduates 77%, and postgraduates 14%). Before completing the online survey, every participant was told of the broad nature of the study. After reading the study information, participants signed an informed consent form that included the study protocol and procedure in accordance with the Declaration of Helsinki. The protocol was approved by the ethics board at the Faculty of Psychology, Beijing Normal University.

### Measures

All measures were carried out in Chinese. In order to reduce the sequence effects, we randomly presented the order of the items in each questionnaire. All measures originally written in English were translated into Chinese according to standard guidelines (Beaton et al., 2000). For each questionnaire, all items were rated on a 5-point scale with 1 (*strongly disagree*) to 5 (*strongly agree*).

#### Power Contingent Self-Esteem

The relationship between self-esteem and power was assessed with the well-validated 10-item Power Contingent Self-esteem scale (PCSE scale, Wang, 2017, unpublished). The sample items were “When others don’t obey me, it makes me feel really bad” and “Even if others don’t obey me, my feelings of self-worth remain unaffected” (reverse scored; please see details in **Appendix**). As well as Knee et al. (2008)’s relationship contingent self-esteem scale used 1–5 scale, we instructed participants to “indicate the extent to which you agree or disagree with each of the item” on a 5-point scale with 1 (*strongly disagree*) to 5 (*strongly agree*).

Confirmatory factor analysis showed that the one-factor model fit the data well, [χ^2^(25) = 60.59, *CFI* = 0.996, *NFI* = 0.993, *RMSEA* = 0.04, *N* = 853]. Meanwhile, correlation analysis showed PCSE scale had high convergent validity, indexed by negative correlation with autonomy (*r* = −0.27, *p* < 0.0001) and competence (*r* = −0.19, *p* < 0.01) satisfaction, positive correlation with trait anger (*r* = 0.25, *p* < 0.0001) and perceived stress (*r* = 0.53, *p* < 0.0001). Furthermore, multiple regression analysis revealed that PCSE (β = −0.38, *p* < 0.0001) and Knee et al. (2008)’s relationship contingent self-esteem (β = 0.31, *p* < 0.0001) had independent contribution to SWB, which showed PCSE scale showed good discriminant validity from related contingent construct. More important, multiple regression analysis showed that PCSE was the only significant predictor of SWB (β = −0.35, *p* < 0.01) compared with Paradise and Kernis (1999)’s unpublished general contingent self-esteem (β = 0.15, *p* = 0.15), which showed PCSE was a better predictor of SWB than general contingent self-esteem. Its one-month test–retest reliability was 0.65. In the current sample, the Cronbach’s alpha was 0.96.

#### Authenticity

A 12-item Authenticity Scale was used to assess the level of authenticity (Wood et al., 2008). It consisted of three subscales: self-alienation (four items, e.g., “I feel out of touch with the ‘real me’.”), accepting external influences (four items, e.g., “I am strongly influenced by the opinions of others.”), and authentic-living (four items, e.g., “I live in accordance with my values and beliefs.”). The Cronbach’s alpha was 0.87.

#### Subjective Well-Being (SWB)

Life satisfaction was measured with the five-item Satisfaction With Life Scale (SWLS; e.g., “In most ways my life is close to my ideal.”; Diener et al., 1985). The Cronbach’s alpha for the current SWLS measure was 0.88. Positive and negative emotion was gauged with the Positive and Negative Affect Schedule (PANAS; Watson et al., 1988), which is made up of 10 positive and 10 negative adjectives and can be subdivided into the positive affect (PA) and negative affect (NA) subscales. The internal reliabilities were 0.96 for PA and 0.93 for NA. Finally, the score of the SWB was computed by adding the standardized SWLS and PA scores and then deducting the standardized NA score (Sheldon and Elliot, 1999).

#### Socially Desirable Responding

The Social Desirability Response Set-5 (Hays et al., 1989) was used to evaluate the extent to which an individual behaves in a socially approved way. For example, “There have been occasions when I took advantage of someone.” The Cronbach’s alpha was 0.78.

### Results and Discussion

Univariate analyses and correlations among variables are shown in the **Table 1**. The results were consistent with our hypothesis. PCSE was negatively correlated with SWB. Furthermore, multiple regression analysis showed that PCSE emerged as a significant negative predictive variable of SWB (*b* = −0.59, *SE* = 0.16, *p* < 0.01), and this correlation remained significant even after controlling for sex, age, and social desirability (*b* = −0.33, *SE* = 0.14, *p* < 0.01). Furthermore, we found that PCSE predicted authenticity (*b* = −0.40, *SE* = 0.06, *p* < 0.01), which held even after controlling for sex, age, and social desirability (*b* = −0.31, *SE* = 0.06, *p* < 0.01).

**Table 1:** Descriptive statistics and correlation among variables (*N* = 210, Study 1).

	1	2	3	4	5	6	7	8
(1) Education	−							
(2) Income	0.345^∗∗∗^	1						
(3) Gender	−0.076	0.012	−					
(4) Age	−0.124	0.257^∗∗∗^	0.19^∗∗^	−				
(5) Social desirability	0.120	0.214^∗∗^	−0.05	0.01	−			
(6) Power contingent self-esteem	0.038	−0.068	−0.05	−0.08	−0.21^∗∗^	−		
(7) Authenticity	0.152^∗^	0.266^∗∗∗^	−0.06	0.06	0.46^∗∗∗^	−0.40^∗∗∗^	−	
(8) Subjective well-being (SWB)	0.167^∗^	0.400^∗∗∗^	0.01	−0.04	0.56^∗∗∗^	−0.25^∗∗∗^	0.55^∗∗∗^	−
Mean	4.05	4.65	1.57	31.90	0.86	29.27	38.28	0.00
Standard deviations	0.538	1.544	0.50	7.43	1.37	9.51	6.88	2.40

*^∗^p < 0.05, ^∗∗^p < 0.01, ^∗∗∗^p < 0.001.*

Finally, we tested whether authenticity would mediate the relationship between PCSE and SWB using an SPSS macro that is developed to examine mediation models (Preacher and Hayes, 2008). As recommended by Preacher and Hayes (2008), evaluation of the mediation models included analyses of the total and specific indirect effects. Specifically, the parameter estimates, and confidence intervals of the total and specific indirect effects were calculated on 5,000 random samples. Mediation is significant when the confidence intervals of an indirect effect do not include zero. As hypothesized, the 95% percentile CIs of indirect effects of PCSE on SWB through authenticity didn’t include zero, which revealed general authenticity mediated the effects of PCSE on life satisfaction. Furthermore, the mediation effect of authenticity between PCSE and SWB remained significant after controlling for income and education (95% CI = [−0.42, −0.09]). More important, the mediation effects remained consistent across males and females (see **Table 2**).

**Table 2:** Results of mediation models testing whether the effect of power contingent self-esteem (PCSE) on subjective well-being was mediated by authenticity (controlling for gender, age, and social desirability) (*N* = 210, Study 1).

Mediators	Unstandardized regression		Bootstrapping procedure	
	Total effect	Direct effect	Indirect effect	95% CI
PCSE → Authenticity → SWB	−0.33^∗^	−0.05	−0.28^∗^	−0.48 ~ −0.11
PCSE → Authenticity → SWB (Males)	−0.24^∗^	0.01	−0.25^∗^	−0.65 ~ −0.02
PCSE → Authenticity → SWB (Females)	−0.34^∗^	−0.29^∗^	−0.06^∗^	−0.13 ~ −0.01

*PCSE, power contingent self-esteem. ^∗^p < 0.05.*

Besides, to confirm the mediation model of PCSE → authenticity → SWB was robust, we performed another mediation analyses to examine alternative models, specifically whether PCSE mediated the relationship between authenticity and SWB and whether SWB mediated the relationship between PCSE and authenticity. The analysis showed that PCSE did not significantly mediate the relation between authenticity and SWB (95% CI = [−0.10, 0.11]), after controlling for sex, age, social desirability as irrelevant covariates. SWB might mediate the effect of PCSE on authenticity (95% CI = [−0.10, −0.04]), after controlling for sex, age, and social desirability. However, the completely standardized effect size of the indirect effect of PCSE on SWB through authenticity (−0.12) are larger than the indirect effect of PCSE on authenticity through SWB (−0.05). These results confirmed the role of the authenticity in mediating the relation between PCSE and SWB.

All in all, conditional on the model assumption PCSE → authenticity → SWB, our statistical tests showed that authenticity could account for a significant portion of variance, which was consistent with a mediation model.

## Study 2

To confirm the results of Study 1, Study 2 examined whether the PCSE, authenticity, and role satisfaction relationship held constant in various roles—work, romantic, friendship, and parent–child relationships—featured with varying degrees of satisfaction (Heller et al., 2007), and differences in self authenticity (Wang, 2014). According to previous studies, people have different level of authenticity and satisfaction in different relationships (Wang, 2014). For example, people would prefer to express their true ideas with less fear for rejection in close relationship (e.g., friendship relationship), rather than in business relationship (e.g., work) (Clark and Finkel, 2005). However, considering our theoretical hypothesis about a fundamental link between PCSE, authenticity, and life satisfaction, we predicted that role PCSE might be related to role satisfaction, and that the negative effects of role PCSE on role satisfaction would be mediated by role authenticity.

### Participants

Two hundred Chinese participants were recruited from a professional research participation website^2^ (109 women and 91 men; *M*_age_ = 34.37 years, *SD* = 7.54; income ranged from 1000 to 20000 RMB). With the aim of reducing the burden of the participants, all surveys were divided into two parts. All participants completed the first survey and were paid 6 RMB (about $1 U.S.) in May 2016, which was related to the work and romantic domains. One week after the first on-line survey, 112 (56 women and 56 men; *M*_age_ = 35.96 years, *SD* = 7.90; income ranged from 1000 to 20000 RMB) participants responded to second survey and were paid another 6 RMB (about $1 U.S.), which covered friendships and parent-child relationships. After reading the study information, participants signed an informed consent form that included the study protocol and procedure in accordance with the Declaration of Helsinki. The protocol was approved by the ethics board at the Faculty of Psychology, Beijing Normal University.

### Measures

#### Power Contingent Self-Esteem

To measure the extent to which participants’ self-esteem depended on his/her power in a specific role, the instructions of the PCSE scale (Wang, 2017, unpublished) were adjusted according for each role. The role PCSE scale had good reliability for romantic relationships (α = 0.96), friendships (α = 0.94), work (α = 0.95), and parent–child relationships (α = 0.94).

#### Satisfaction in Specific Role

Subjects were asked to respond to each item based on their satisfaction within each concrete role. Satisfaction at work was assessed with Brayfield and Rothe (1951) 5-item scale (α = 0.75). For example, “I feel fairly satisfied with my present job.” and “Most of the time I have to force myself to go to work” (reverse scored; Brayfield and Rothe, 1951). Satisfaction in romantic relationship was assessed with Norton (1983) 6-item romance satisfaction scale (α = 0.90). For example, “My relationship with my romantic partner is very stable” (Norton, 1983). Friendship (α = 0.72) or parent–child relationship (α = 0.77) satisfaction were adapted from the 5-item Hendrick (1988)’s role satisfaction scale. For example, “How well does your friend/parents meet your needs?” (Hendrick, 1988).

#### Authenticity Within Four Specific Roles

We utilized 8-items to assess the extent to which participants experienced authenticity in concrete roles. Three items were chosen from Fleeson and Wilt (2010) authenticity scale (e.g., “I felt like I was really being me”) and 5 items were adapted from the authenticity scale designed by Sheldon et al. (1997; e.g., “I experience this aspect of myself as an authentic part of who I am.” and, “I feel tense and pressured in this part of my life” reverse scored; Sheldon et al., 1997; Fleeson and Wilt, 2010). In the present study, the Cronbach’s alphas were 0.81 working relationships, 0.78 for romantic relationships, 0.82 for friendships, and 0.87 for parent-child relationships, respectively.

### Results

The descriptive analysis for role PCSE, role satisfaction, and role authenticity within the four roles are shown in **Table 3** and correlations between the three variables across the four roles are presented in **Table 4**. As we predicted, an analysis of variance (ANOVA) showed that there were significant differences in participants’ role satisfaction [*F*(3,109) = 8.31, *p* < 0.001] and authenticity [*F*(3,109) = 42.48, *p* < 0.001], but not role PCSE [*F*(3,109) = 1.75, *p* = 0.16]. Specifically, participants reported the highest satisfaction in romantic relationships and most authenticity in parent–child relationships.

**Table 3:** Descriptive for power contingent self-esteem (PCSE), authenticity, and satisfaction within four roles (Study 2).

	Work (*N* = 200)			Romantic-relationship (*N* = 200)			Friendship (*N* = 112)			With parents (*N* = 112)		
	α	Mean	*SD*	α	Mean	*SD*	α	Mean	*SD*	α	Mean	*SD*
Role PCSE	0.95	25.64	9.00	0.96	27.14	9.56	0.94	24.17	8.29	0.94	24.50	8.77
Subjective well-being	0.68	3.85	0.61	0.90	4.19	0.63	0.72	4.03	0.52	0.77	4.24	0.57
Role authenticity	0.81	30.28	4.57	0.78	28.06	3.40	0.82	33.04	3.75	0.87	33.62	4.61

*PCSE, power contingent self-esteem.*

**Table 4:** Correlations between role power contingent self-esteem (PCSE) and satisfaction within four roles (Study 2).

	Work (*N* = 200)		Romantic-relationship (*N* = 200)		Friendship (*N* = 112)		With parents (*N* = 112)	
	2	3	2	3	2	3	2	3
(1) Role PCSE	−0.31^∗∗∗^	−0.37^∗∗∗^	−0.24^∗∗∗^	−0.34^∗∗∗^	−0.39^∗∗∗^	−0.52^∗∗∗^	−0.41^∗∗∗^	−0.38^∗∗∗^
(2) Role satisfaction		0.71^∗∗∗^		0.77^∗∗∗^		0.79^∗∗∗^		0.80^∗∗∗^
(3) Role authenticity		−		−		−		−

*PCSE, power contingent self-esteem. ^∗∗∗^p < 0.001.*

Second, regression analysis across the four role surveys verified our hypothesis that the negative association between PCSE on SWB can be generalized to various contexts, as role PCSE was significantly correlated with role satisfaction in all four roles (controlling for sex, age, and social desirability; **Table 5**).

**Table 5:** Results of multiple regressions for the prediction of role satisfaction (Study 2).

Predictor	Romantic-relationship (*N* = 200)		Work (*N* = 200)		Friendship (*N* = 112)		Parents–child (*N* = 112)	
	Model 1	Model 2	Model 1	Model 2	Model 1	Model 2	Model 1	Model 2
Sex	−0.04	−0.04	−0.01	0.01	0.06	0.04	−0.01	0.01
Age	−0.11	−0.10^∗^	0.04	0.01	0.01	−0.02	−0.02	0.12
Social desirability	0.33^∗∗∗^	0.10^∗^	0.32^∗∗∗^	0.13^∗^	0.29^∗∗^	0.09	0.14	0.04
PCSE	−0.16^∗^	0.03	−0.21^∗∗^	−0.03	−0.28^∗∗^	0.04	−0.37^∗∗∗^	−0.10
Role authenticity		0.73^∗∗∗^		0.65^∗∗∗^		0.78^∗∗∗^		0.77^∗∗∗^
*R*^2^	0.43	0.78	0.43	0.72	0.47	0.79	0.43	0.81
Δ*R*^2^		0.35		0.29		0.32		0.38

*PCSE, Power-Contingent Self-Esteem; For each role, Model 1 tested the direct effect of role PCSE on role satisfaction; Model 2 tested whether the inclusion of authenticity in the regression model reduced the effects of PCSE on role satisfaction. ^∗^p < 0.05, ^∗∗^p < 0.01, ^∗∗∗^p < 0.001.*

Third, role PCSE emerged as a negative predictive variable of role authenticity across each of four roles: working relationships (*b* = −0.28, *SE* = 0.07, *p* < 0.001), romantic relationships (*b* = −0.26, *SE* = 0.07, *p* < 0.001), friendship relationships (*b* = −0.42, *SE* = 0.09, *p* < 0.001), parent–child relationships (*b* = −0.35, *SE* = 0.09, *p* < 0.001); again, these effects held after controlling for sex, age, social desirability, and income (*p*s < 0.05). At last, mediation analysis using Preacher and Hayes (2008) SPSS macro showed that authenticity mediated the association between PCSE and satisfaction in four concrete roles (see **Table 6**).

**Table 6:** Results of mediation analyses testing whether the effect of power contingent self-esteem (PCSE) on role satisfaction was mediated by authenticity (controlling of sex, age, and social desirability, Study 2).

Proposed mediation models	Unstandardized regression		Bootstrapping procedure	
	Total effect	Direct effect	Indirect effect	95% CI
Work: PCSE → authenticity → satisfaction	−0.21^∗∗∗^	−0.03	−0.18^∗^	−0.33 ~ −0.05
Romantic relationship: PCSE → authenticity → satisfaction	−0.16^∗^	0.03	−0.19^∗^	−0.33 ~ −0.08
Friendship: PCSE → authenticity → satisfaction	−0.28^∗∗^	0.04	−0.32^∗∗^	−0.54 ~ −0.16
Parent-Child: PCSE → authenticity → satisfaction	−0.37^∗∗∗^	−0.10	−0.27^∗∗^	−0.46 ~ −0.13

*PCSE, power contingent self-esteem. ^∗^p < 0.05, ^∗∗^p < 0.01, ^∗∗∗^p < 0.001.*

Then, we divided participants into two groups according to gender and performed mediation analyses within the two groups, respectively. The mediation analyses showed that the mediation effects of role authenticity between role PCSE and role satisfaction didn’t differ from males to females (see **Tables 7, 8**).

**Table 7:** Results of mediation analyses testing whether the effect of power contingent self-esteem (PCSE) on role satisfaction was mediated by authenticity in the group of males (Study 2).

Proposed mediation models	Unstandardized regression		Bootstrapping procedure	
	Total effect	Direct effect	Indirect effect	95% CI
Work: PCSE → authenticity → satisfaction	−0.52^∗∗∗^	−0.14	−0.37^∗∗∗^	−0.60 ~ −0.17
Romantic relationship: PCSE → authenticity → satisfaction	−0.35^∗∗∗^	−0.03	−0.32^∗∗∗^	−0.52 ~ −0.11
Friendship: PCSE → authenticity → satisfaction	−0.46^∗∗^	−0.12	−0.34^∗∗^	−0.61 ~ −0.15
Parent–child: PCSE → authenticity → satisfaction	−0.46^∗∗∗^	−0.23^∗^	−0.23^∗^	−0.46 ~ −0.06

*PCSE, power contingent self-esteem. ^∗^p < 0.05, ^∗∗^p < 0.01, ^∗∗∗^p < 0.001.*

**Table 8:** Results of mediation analyses testing whether the effect of power contingent self-esteem (PCSE) on role satisfaction was mediated by authenticity in the group of females (Study 2).

Proposed mediation models	Unstandardized regression		Bootstrapping procedure	
	Total effect	Direct effect	Indirect effect	95% CI
Work: PCSE → authenticity → satisfaction	−0.16	−0.01	−0.15^∗^	−0.34 ~ −0.02
Romantic relationship: PCSE → authenticity → satisfaction	−0.17	0.05	−0.22^∗^	−0.41 ~ −0.07
Friendship: PCSE → authenticity → satisfaction	−0.35^∗∗∗^	0.13	−0.48^∗∗∗^	−0.80 ~ −0.23
Parent–child: PCSE → authenticity → satisfaction	−0.34^∗∗∗^	−0.02	−0.36^∗∗∗^	−0.57 ~ −0.15

*PCSE, power contingent self-esteem. ^∗^p < 0.05, ^∗∗^p < 0.01, ^∗∗∗^p < 0.001.*

Besides, to confirm the current mediation model, we performed alternative mediation analyses to examine whether role PCSE mediated the relationship between role authenticity and satisfaction and whether role satisfaction mediated the relationship between role PCSE and authenticity. The analysis showed that role PCSE did not significantly mediate the relation between role authenticity and role satisfaction in work (95% CI = [−0.03, 0.07]), romantic relationship (95% CI = [−0.05, 0.02]), friendship (95% CI = [−0.06, 0.04]), and parent-child (95% CI = [−0.01, 0.07]). Role satisfaction mediated the effects of role PCSE on authenticity at work (95% CI = [−0.25, −0.03]), romantic relationship (95% CI = [−0.31, −0.08]), friendship (95% CI = [−0.19, −0.08]), and parent–child relationship (95% CI = [−0.15, −0.05]), after controlling for age and social desirability. However, the completely standardized effect sizes of indirect effects of role PCSE on role satisfaction through authenticity in the roles of romantic relationship (−0.30) and friendship (−0.41) are larger than that in the roles of romantic relationship (−0.26) and friendship (−0.27). These results further confirmed the role of the authenticity in mediating the relation between PCSE and SWB.

All in all, study 2 further confirmed the finding of Study 1, showing that if role authenticity was to be included in a regression model, it would absorb a significant part of the variance shared between role PCSE and role satisfaction.

## Discussion

The purpose of the current study was to examine whether and how a form of self-esteem extremely depended on power—PCSE—was linked to individual’s SWB. The result of Study 1 showed that general PCSE was consistently linked to low SWB. More importantly, the negative relationship between PCSE and SWB was mediated by lower experienced authenticity. Study 2 further confirmed the mediation effect between role PCSE and role satisfaction through authenticity across four different roles (work, romance, friendship, and parent–child relationships). The finding that the negative association between PCSE and SWB is mediated by the authenticity contributes to uncovering the complicated relationships between power, self-esteem, and life satisfaction and has important theoretical and practical implications.

First, we proposed a new type of power contingent self-esteem which assesses extent to which people extremely depend their self-esteem on power. It will move the field of contingent self-esteem and power contingency with emphasizing the negative sides of extreme contingency. Furthermore, it will contribute to revealing why contingent self-esteem sometimes shows negative effects, but sometimes not (Crocker et al., 2002). Meanwhile, how different kinds of contingent self-esteem (i.e., social approval, appearance, or academic) jointly influence people’s well-being remains an interesting issue for future research to explore.

Second, out results are consistent with previous findings showing that contingent self-esteem is a negative predictor for well-being (Deci et al., 1999). Furthermore, our results expanded existing findings (Paradise and Kernis, 1999, unpublished; Knee et al., 2008) by showing that when people are less true to their inner desires and inclinations, PCSE makes people to feel less pleasure. Contributing to the existing literature on contingent self-esteem and SWB, the current findings reveal the mechanism underlying the relationship between PCSE and well-being. Once self-esteem is extremely contingent on power, it will be associated with inauthentic and extrinsic motivation, which is then associated with lower life satisfaction. In particular, individuals reported higher authenticity and satisfaction in close relationships, such as friendships, rather than in business relationships (Clark and Finkel, 2005). Nonetheless, role authenticity mediates the relationship between role PCSE and satisfaction within all four specific roles.

Third, we realized that the high correlations between role authenticity and role satisfaction in Study 2 made it hard to test which variable is the true mediator using statistical mediation tests. However, alternative models, such as SWB mediates the relationship between PCSE and authenticity, might make less sense conceptually. According to mainstream counseling psychology perspectives, authenticity has seen as the most fundamental aspect of well-being (Horney, 1951; Winnicott, 1965; May, 1981), which builds the theoretical foundation of the current study. By contrast, it remains unclear how being satisfied would cause someone to feel more authentic. Furthermore, we found that the models with authenticity as the mediator produce larger effect sizes in Study 1 and two cases of Study 2 which supported our theoretical speculation.

Four, based on current findings, future intervention-based research might help people to promote authenticity well-being through lowering PCSE. For example, initial assessment using PCSE scale might help people to identify whether they have depended their self-esteem on power to an extreme extent. Then, interventions might focus people on lowering their contingencies on sense of power. Specific interventions might include cognitive and insight therapies to enhance people acknowledge on difference between real self-esteem and contingent self-esteem; behavioral and skills training to separate self-esteem from personal power in specific roles.

Several limitations of our research warrant attention. At first, although our statistical tests showed that authenticity could account for a significant portion of variance that is consistent with a mediation model, it is also compatible with several other models which cannot be fully ruled out by reverse mediation testing approach. Hence, future studies are suggested to repeatedly test the assumed causal process with sound theorizing and various strategies to help the “true” indirect effect to stand up (Lemmer and Gollwitzer, 2017; Fiedler et al., 2018). Second, all our subjects were from Chinese culture, and thus, whether the current findings are generalizable to Western cultures should be examined in future studies. Third, all our measures are self-reported data. Behavioral measurement or other-reported data are needed to examine the generalizability of our results using various methods. Four, future longitudinal studies are preferred for purpose of better illustrating the causal relationship among power, self-esteem, and SWB.

The current research suggests that, although both power and self-esteem make for well-being, self-esteem that is extremely contingent on power will be detrimental to well-being through lower authenticity. To maintain well-being, it is necessary to free the self from a sense of power, which is a prerequisite for true self-esteem.

## Author Contributions

YW designed the study, collected and analyzed the data, and wrote the paper. ZL wrote the paper.

## Conflict of Interest Statement

The authors declare that the research was conducted in the absence of any commercial or financial relationships that could be construed as a potential conflict of interest.

## Footnotes

1 http://www.sojump.com/

2 http://www.sojump.com/

## Appendix

**Table A1:** Final power-contingent self-esteem scale with corrected item-total correlations (*N* = 853).

Items	Item-total correlation
(1) When others don’t obey me, it makes me feel really bad.	0.85
(2) When my ideas have no influence on others, I feel bad about myself in general.	0.85
(3) When others don’t value my opinions, I feel bad about myself.	0.88
(4) When my opinion is ignored, my feelings of self-worth are damaged.	0.87
(5) When my decision can’t affect others, I have poor self-feelings.	0.83
(6) Even if others don’t obey me, my feelings of self-worth remain unaffected.	0.84
(7) Even if others care nothing about my opinions, my self-worth is unaffected.	0.83
(8) Even if others ignore my thoughts, I would not let it affect how I feel about myself.	0.83
(9) Even if others don’t take my advice, my feelings of self-worth are not affected.	0.84
(10) Even if my opinions count for little to others, I will not feel bad about myself.	0.80

*N = 853. Items are rated on a scale from 1 to 5, with anchors of 1 (not at all like me), 3 (somewhat like me), and 5 (very much like me).*
